# Epidermal stem cell-derived exosomes promote skin regeneration by downregulating transforming growth factor-β1 in wound healing

**DOI:** 10.1186/s13287-020-01971-6

**Published:** 2020-10-23

**Authors:** Mengna Duan, Yan Zhang, Haiyang Zhang, Yupeng Meng, Ming Qian, Guokun Zhang

**Affiliations:** 1 Department of Prosthodontics, Hospital of Stomatology, Jilin University, 1500 Qinghua Rd., Changchun, 130021 Jilin China; 2 Jilin Provincial Laboratory of Biomedical Engineering, Jilin University, 1500 Qinghua Rd., Changchun, 130021 Jilin China; 3 Institute of Antler Science and Product Technology, Changchun Sci-Tech University, 1345 Pudong Rd., Changchun, 130600 Jilin China; 4 Institute of Special Animal and Plant Sciences, Chinese Academy of Agricultural Sciences (CAAS), 4899 Juye St., Changchun, 130112 Jilin China

**Keywords:** Exosome, Epidermal stem cells, MicroRNA, Myofibroblast, Transforming growth factor-β1

## Abstract

**Background:**

Scar formation, which may be caused by myofibroblast aggregations, is the greatest challenge during skin wound healing in the clinical setting. Studies have indicated that epidermal stem cells (EPSC) improve wound healing and reduce scar formation.

**Methods:**

We investigated the therapeutic effects of EPSC-derived exosomes (EPSC-Exos) on skin wound healing in a skin-defect rat model. We also examined the roles of EPSC-Exos-specific microRNAs in inhibiting the differentiation of human dermal fibroblasts (HDF) into myofibroblasts.

**Results:**

We found that EPSC-Exos increased the wound healing rate and reduced scar formation in rats. Also, EPSC-Exos improved the regeneration levels of skin appendages, nerves and vessels, as well as the natural distribution of collagen. Furthermore, we found these functions may be achieved by inhibiting the activity of transforming growth factor-β1 (TGF-β1) and its downstream genes. The results showed that some specific microRNAs, including miR-16, let-7a, miR-425-5p and miR-142-3p, were enriched in EPSC-Exos. EPSC-Exos-specific microRNAs, especially miR-425-5p and miR-142-3p, played vital roles in inhibiting myofibroblast differentiation via reducing the TGF-β1 expression in dermal fibroblasts.

**Conclusion:**

We found a novel function of EPSC-Exos-specific microRNAs, suggesting that EPSC-Exos might represent a strategy to prevent scar formation during wound healing in the clinical setting.

## Background

The skin is a multilayer interface between the body and the environment, which can regulate temperature, prevent dehydration, keep out pathogens, provide sensation and transport water [1–3]. Once the skin is damaged, the body will immediately start a repair programme to complete the recovery of the skin’s structure and function. However, fibrosis or scarring during the healing process cannot be avoided, even under optimal conditions [3, 4]. Many approaches have attempted to achieve the best healing result, namely the double reconstruction of structure and function. Stem cell-based therapy, with unique advantages, is emerging as a promising candidate for wound healing and skin regeneration [5, 6]. Epidermal stem cells (EPSC) are a type of autologous adult stem cells that are easily obtainable from the skin. EPSC reportedly differentiated into hair follicles and sweat glands for use in tissue repair, decreasing scar formation and providing long-term regeneration [7–11]. However, there is only a small amount of EPSC in the healing tissue after EPSC treatment. According to the mainstream view [12], the strong paracrine ability of EPSC may be the essential mechanism resulting in these therapeutic effects.

Exosomes, secreted by parental cells, are a type of membrane structure, encapsulating various mRNAs, miRNAs, LncRNAs, CircRNA and proteins to regulate the activity of recipient cells. Exosomes play important roles in tissue regeneration [13–15]. They have a similar function to their parent cells, but they are easier to store and transport, protecting the bioactive substances they carry from adverse conditions such as pH environments, high temperature and repeated freezing and thawing [16–18]. Moreover, they avoid many of the risks associated with cell transplantation. Therefore, exosome therapy may be a more safe and efficient option.

Transforming growth factor-β1 (TGF-β1) is the primary regulator in wound healing and tissue repair. It is responsible for cellular proliferation, differentiation and metabolism [3, 19]. Excessive scar formation and fibrosis are closely related to high expression levels of TGF-β1 in wound healing [1, 3, 20]. TGF-β1 induces the differentiation of myofibroblasts via the phosphorylation of the SMAD family of proteins and activating downstream-related genes such as α-smooth muscle actin (α-SMA) and collagen I [3, 19, 20]. Therefore, inhibiting the activities of TGF-β1 and its downstream genes might reduce myofibroblast formation and over-aggregation to decrease scarring.

In the current study, we investigated whether EPSC-derived exosomes (EPSC-Exos) are effective in the healing of cutaneous wounds using full-thickness skin-defect rat models. Moreover, we identified two specific microRNAs carried by EPSC-Exos as critical components contributing to the suppression of the differentiation of fibroblasts to myofibroblasts by inhibiting TGF-β1 expression. Thus, the results suggest that EPSC-Exos treatment could be a potential strategy to prevent excessive fibrosis and scar formation during wound healing in injured skin.

## Materials and methods

### Cell culture

The cell lines of EPSC and human dermal fibroblasts (HDF) were obtained from the China-Japan Union Hospital of Jilin University. Cells were cultured in Dulbecco’s modified Eagle medium (+ 10% foetal bovine serum; Biological Industries, Beit-Haemek, Israel) with 5% CO_2_ at 37 °C. Cells at the 3–5 passages were used for this study.

### Exosome isolation

EPSC-Exos were purified by ultracentrifugation according to previously reported methods [15, 18]. Briefly, EPSC were cultured in a serum-free medium (Gibco, Grand Island, NY) for 48 h. The culture suspension was filtered (0.1-μm filter device) and concentrated (100-kDa molecular weight cutoff), then loaded onto a 30% sucrose/deuterium oxide (D2O) cushion and ultracentrifuged at 100,000×*g* for 3 h. The exosomes were then washed with phosphate-buffered saline (PBS) 3 times and centrifuged at 1500×*g* for 30 min (100-kDa molecular weight cutoff). The protein concentration of the exosomes was determined using a bicinchoninic acid protein assay kit (Solarbio, Beijing, China). The exosomes were stored at − 80 °C.

### Creation of skin injury model in rats and treatment with EPSC-Exos

The Animal Experimental Ethics Committee’ of Jilin University approved all study protocols and procedures (Approval No. SY201902010). Thirty Sprague Dawley rats (8-week-old, female, 200 g) were purchased from Liaoning Changsheng Biotechnology Co., Ltd. (Benxi, China) and reared under standard experimental animal feeding conditions. The rats were anaesthetised with 10% chloral hydrate (3 ml/kg), after which their hair was removed from the dorsal surface. A 12-mm diameter was removed to establish a full-thickness skin-defect model.

EPSC-Exos was dissolved into HydroMatrix (Sigma-Aldrich, St. Louis, MO) used as a scaffold. Briefly, 100 μg/ml of EPSC-Exos dissolved in PBS and 10 mg/ml (1%) hydrogel mixed at a ratio of 1:1 and injected (200 μl) around the wound once a week for 4 weeks. Skin damage was recorded photographically every 7 days. The area of each wound was calculated out using Adobe Photoshop CS6. Firstly, lasso tool was used to trace the edge of a wound on a photograph and to circle it; then, we calculate the circled area based on the pixels of that area (1 cm = 28.346 pixels). PBS alone served as a negative control, and epidermal growth factor (EGF; Beyotime, Shanghai, China) mixed with hydro-matrix served as a positive control, both PBS and EGF + hydro-matrix were also injected around the wound once a week for 4 weeks.

The rats were euthanised after being anaesthetised with 10% chloral hydrate (3 ml/kg), and the healing wound tissues collected for later use.

### Histopathological analysis

Skin tissue was embedded in paraffin, then sliced into 4-μm sections. The sections were stained with haematoxylin and eosin (Solarbio, Beijing, China), Masson (Solarbio), immunohistochemistry (IHC), following the manufacturer’s instructions and conventional methods. The primary antibodies used in IHC were anti-CD31 (bs-0468R, 1:500 dilution; Bioss, Beijing, China), anti-Nestin (bs-0008R, 1:500 dilution; Bioss) and Ki67 (bs-23105R, 1:500 dilution; Bioss). Sections were photographed using the Digital Imaging Scanning System (Precipoint M8; Precipoint, Freising, Germany). The Masson staining and IHC results were analysed using Image-Pro Plus software. Firstly, we counted the area of the target item in the picture and then calculated its ratio to the total area of the picture.

### Co-culture system of EPSC and HDF

The 5 × 10^4^ HDF and ESCs were seeded with Dulbecco’s modified Eagle medium and 10% foetal bovine serum on the upper (0.4 μm) and lower chambers of 24-well plates (Corning Life Sciences, NY). After incubating plates for 48 h at 37 °C, TGF-β1 expression levels of HDF were performed using quantitative immunofluorescence (IF) staining and quantitative real-time polymerase chain reaction (qRT-PCR).

### qRT-PCR

Trizol reagent (Tiangen, Beijing, China) was used for total RNA isolation of the skin tissue. cDNA was prepared via reverse transcription of RNA. cDNA, primers, and SYBR premix (Roche, Basel, Switzerland) were combined on qRT-PCR using the ABI 9700 Detection System (Thermo Fisher Scientific, Waltham, MA), and GAPDH was used as control. Supplemental Table S1 shows the primers. All experiments were repeated three times. qRT-PCR for miRNA was done using miScript SYBR Green PCR kit (Qiagen, Hiden, Germany) according to the manufacturer’s instruction. All results were normalised to U6 small RNA levels measured using the Hs_RNU6B_2 miScript Primer Assay kit (Qiagen). Supplemental Table S1 shows the primers. All experiments were repeated three times.

### IF staining

HDF with a density of 70–80% were incubated for 10 min with 10% formaldehyde and for 30 min with 1% bovine serum albumin (Beyond, Shanghai, China). Next, the cells were incubated with anti-TGF-β1 (BSM-33345M, 1:500 dilution; Bioss) at 4 °C overnight and incubated with anti-IgG (A0408, 1:500 dilution) at 25 °C for 30 min. The nucleus was labelled with 4′,6-diamidino-2-phenylindole (Beyotime, Shanghai, China).

### Statistical analysis

All quantitative data were expressed as the mean ± SD. One-way analysis of variance with Tukey’s multiple comparisons test was used for statistically significant differences. Results were considered significant at a *P* value of less than 0.05.

## Results

### EPSC-Exos improve wound healing rate and suppress scar formation

**Fig. 1:**
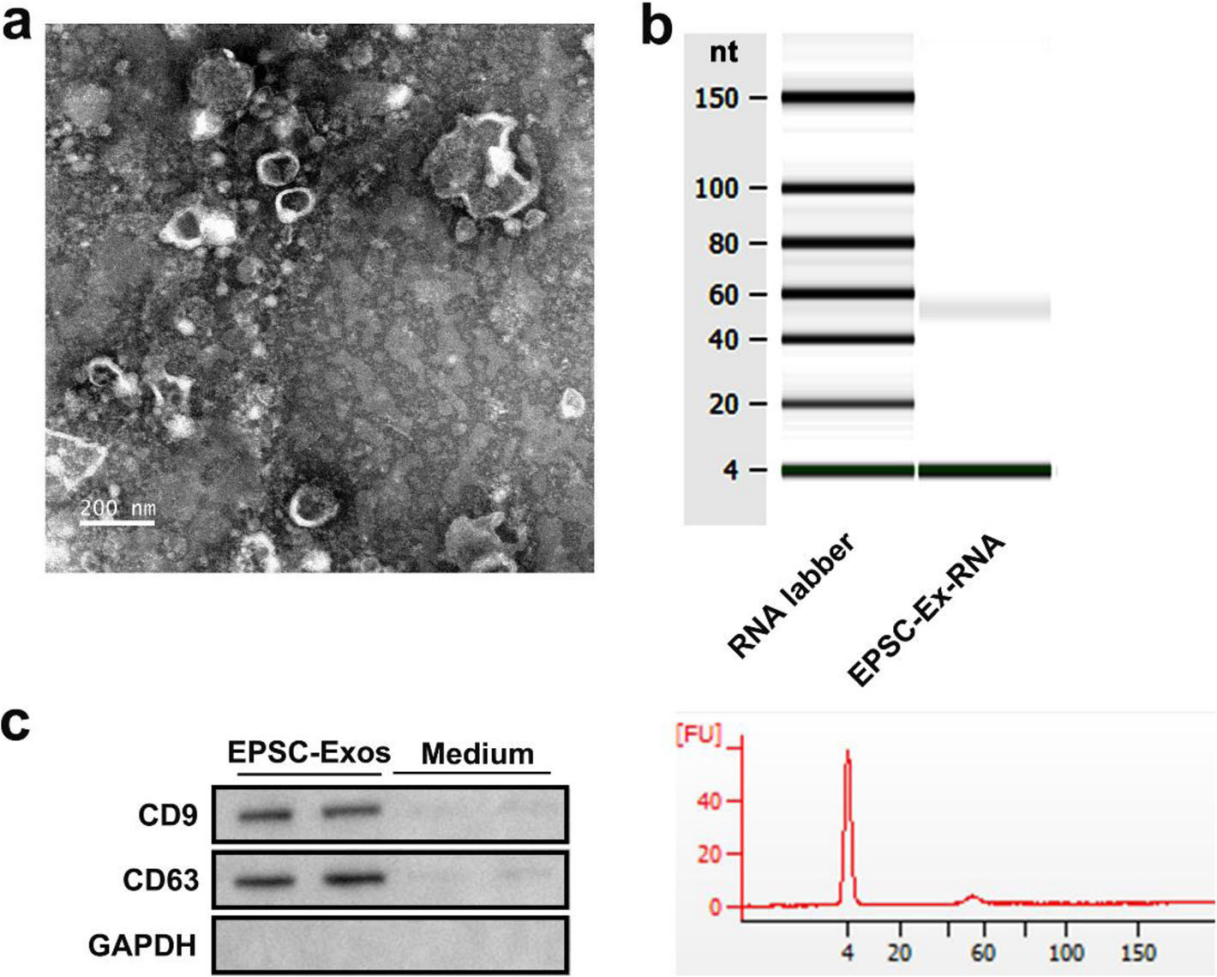
Characterisation of exosomes derived from EPSC. EPSC-Exos were isolated and purified from EPSC supernatant using high-speed centrifugation. **a** Identification of the main morphological characteristics of EPSC-Exos by transmission electron microscopy images. Scale bar = 100 nm. **b** RNA expression profiles of EPSC-Exos by Genechip analysis. **c** The markers of exosomes are detected using western blot assay, and the cell-free medium was added as a control. EGF, epidermal stem cells; EPSC-Exos, epidermal stem cell-derived exosomes

We first identified the purified EPSC-Exos. Transmission electron microscopy results showed that the particle size of EPSC-Exos was between 30 and 100 nm (Fig. 1a). The RNA in EPSC-Exos was analysed via agarose gel electrophoresis. There was only a clear band at 60 nt (Fig. 1b). Analysis using the Agilent 2100 Bioanalyzer confirmed these results (Fig. 1b). Western blot analysis was used to detect the expression of exosomal markers CD9 and CD63, and serum-free medium was used as a control. The results showed that CD9 and CD63 were abundant in EPSC-Exos (Fig. 1c). In summary, the isolated EPSC-Exos has high purity and can be used in subsequent experiments.

**Fig. 2:**
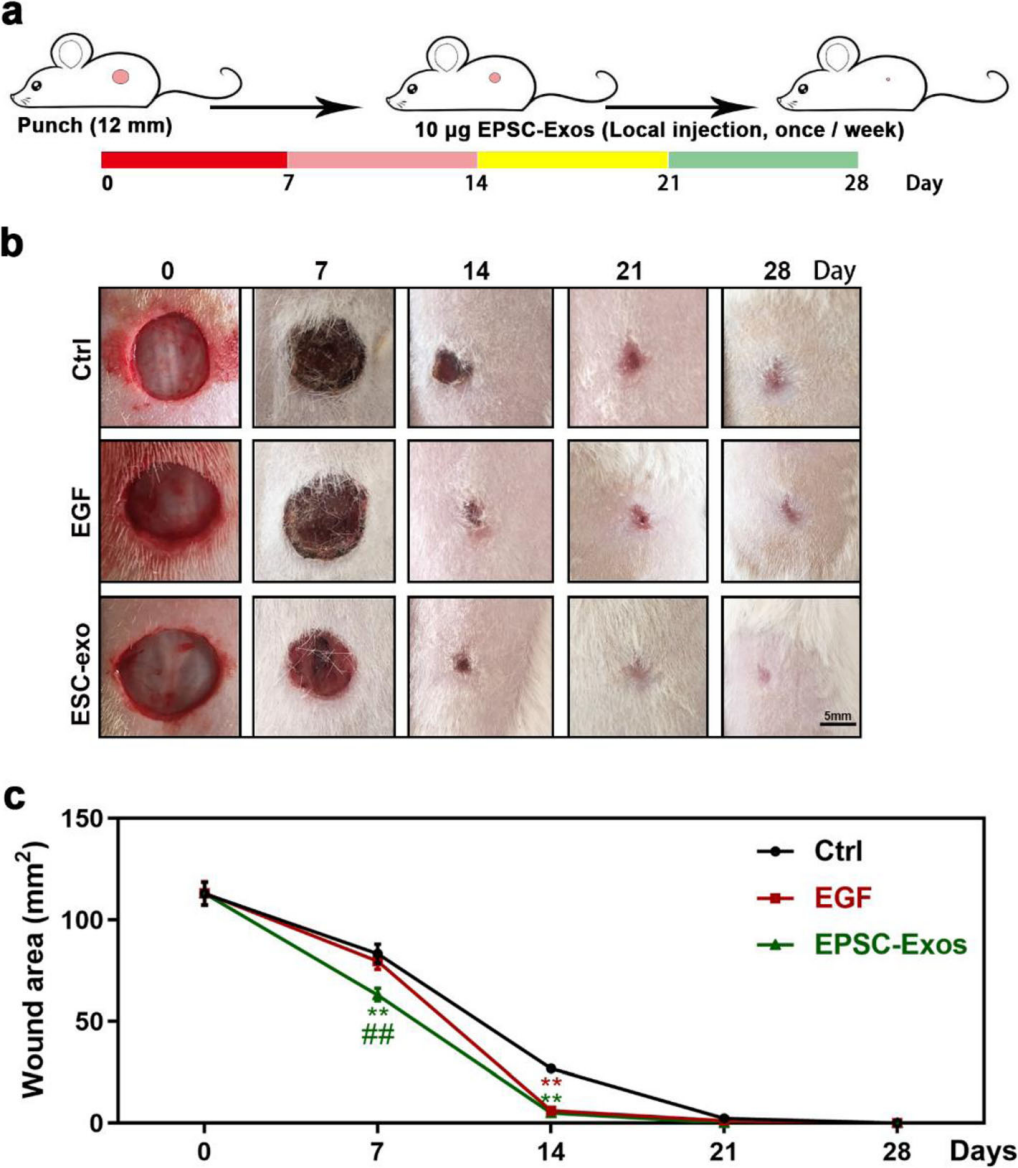
EPSC-Exos increased the wound healing rate and suppressed scar formation in a full-thickness skin-defect rat model. **a** Experimental procedure. The dorsal area of the rats was shaved under anaesthesia. Then, circular hole (12-mm diameter), full-thickness skin excisional wounds were made on the shaved skin. The rats were randomly divided into three groups (*n* = 10/group): phosphate-buffered saline (PBS, negative control), epidermal growth factor (EGF, positive control, 10 μg), and EPSC-Exos (positive control; 10 μg). The rats were treated via local injection around the wound once a week for 4 weeks, and skin damage was recorded photographically every 7 days. **b** Overall morphological changes observed during the wound healing. Scale bar = 5 mm. **c** Changes in wound area during healing. **P < 0.01 compared with the control; ^##^*P* < 0.01 compared with EGF; mean ± SD. Ctrl, control; EGF, epidermal growth factor; EPSC-Exos, epidermal stem cell-derived exosomes

To clarify the effects of EPSC-Exos in the wound healing rate and scar formation, we used full-thickness skin-defect rats and injected equal quantities of hydrogel-coated EPSC-Exos, PBS or EGF around the wounds. The results are shown in Fig. 2. After 1 or 2 weeks of treatment, the wound areas and scars of the EPSC-Exos group were smaller than those of the EGF and control groups. After 3 weeks, the defects in the EPSC-Exos group were closed, but those in the EGF and control groups were not. After 4 weeks, the defects in the EGF and control groups were also closed. However, they exhibited more scar formation than that observed in the EPSC-Exos group. These results suggest that EPSC-Exos can improve the wound healing rate and suppress scar formation.

### EPSC-Exos improve the regeneration level of skin appendages and collagen distribution on wound healing of rats

**Fig. 3:**
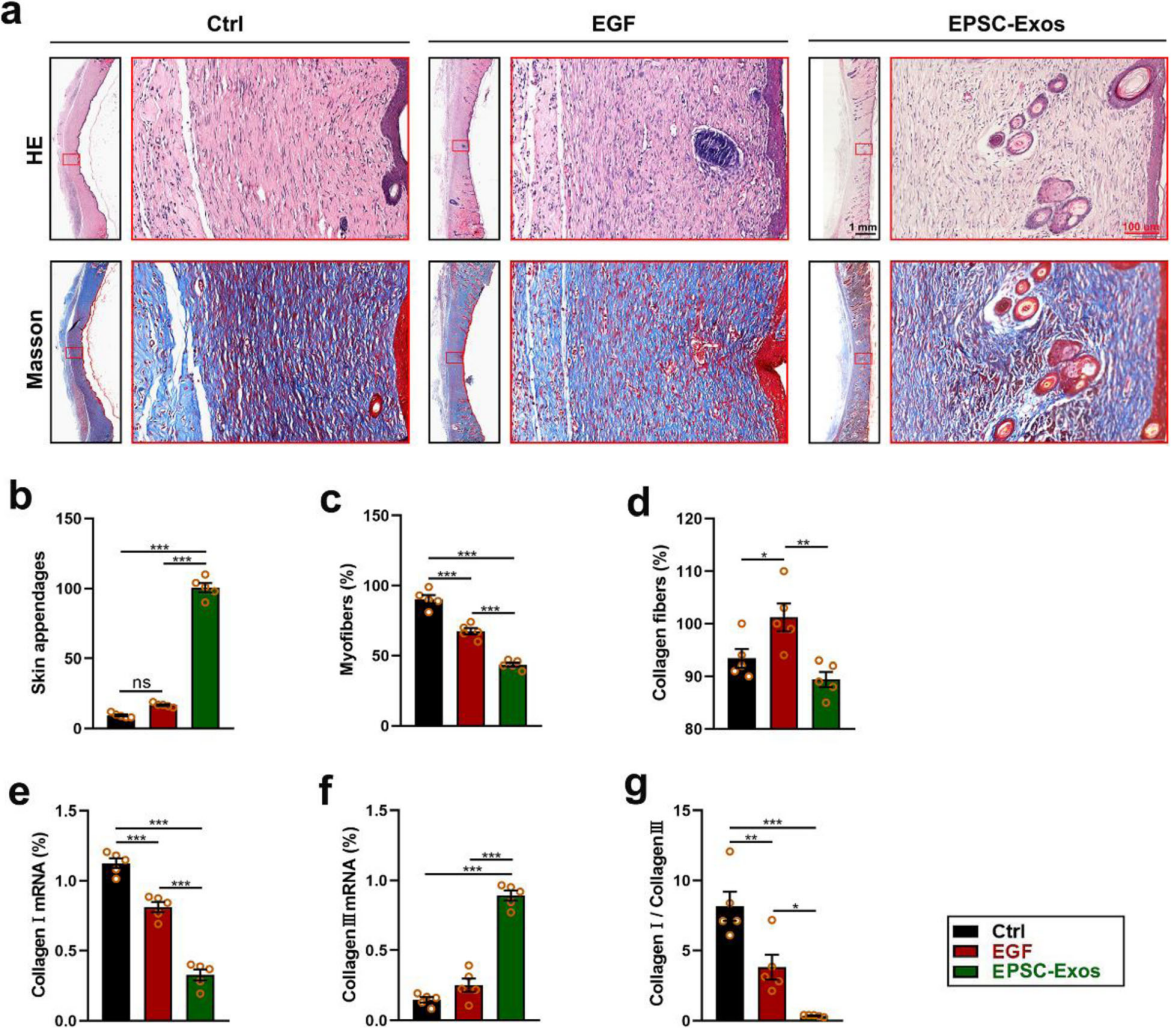
EPSC-Exos improved the regeneration level of skin appendages and the collagen distribution on wound healing of the rats. **a** Haematoxylin and eosin and Masson staining of the wound healing tissues. Scale bar = 100 μm. **b** Number of skin appendages (hair follicles and sebaceous glands) in the healing tissue according to haematoxylin and eosin staining. **c**, **d** The proportion of myofibers (red) and collagen fibres (blue) in the healing tissue, according to Masson staining. **e**–**g** Collagen I mRNA, collagen III mRNA and collagen I/collagen III levels in the healing tissue. *P < 0.05; **P < 0.01; ***P < 0.001; mean ± SD; *n* = 5. Ctrl, control; EGF, epidermal growth factor; EPSC-Exos, epidermal stem cell-derived exosomes

After evaluating the effect of EPSC-Exos on increasing the wound healing rate and decreasing scar formation in shape and appearance, we made further evaluations at the histological level. As shown in Fig. 3a and b, the results showed that after 4 weeks of treatment, the skin in the EPSC-Exos group exhibited more appendages than that in the EGF and control groups (significant; *P* < 0.001). There were significantly fewer myofibers (red) in the EPSC-Exos group than the EGF and control groups (*P* < 0.001) (Fig. 3a, c). In addition, there were significantly fewer collagen fibres (blue) in the skin of the EPSC-Exos group than observed in the EGF group (*P* < 0.01) (Fig. 3a, d). Moreover, among the regenerated collagen fibres, the rates of collagen I and III in the EPSC-Exos group were lower than in the EGF and control groups (Fig. 3e–g). These results suggest that EPSC-Exos can promote skin appendage regeneration, decrease myofiber formation and increase collagen III in wound healing.

### EPSC-Exos promote nerve and vessel regeneration on wound healing in rats

**Fig. 4:**
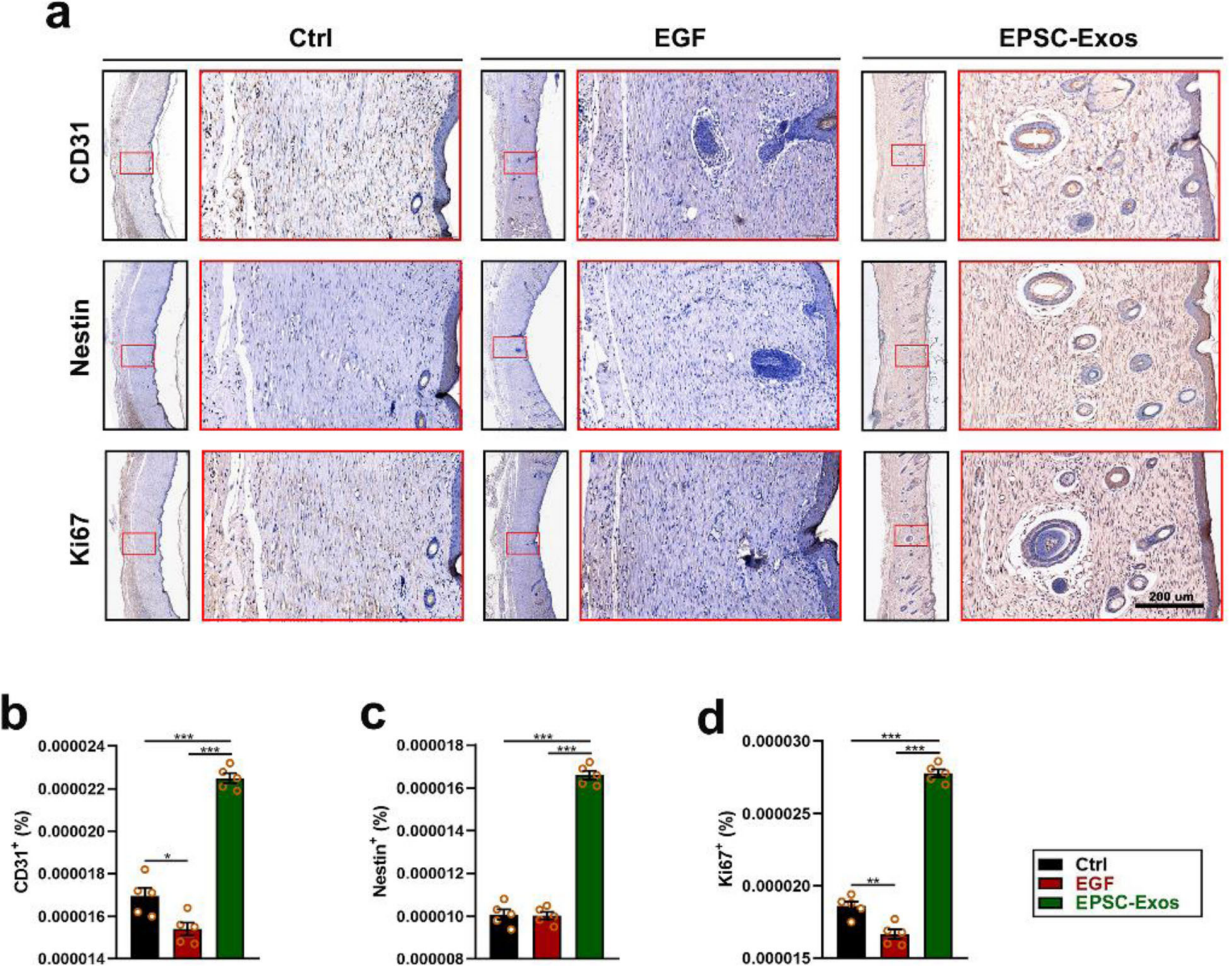
EPSC-Exos promote nerve and vessel regeneration on wound healing of rats. **a** Immunohistochemistry of the wound healing tissues. Scale bar = 200 μm. **b**–**d** The proportion of CD31^+^ (vessel), nestin^+^ (nerve) and Ki67^+^ (cell proliferation) expressions in the healing tissue according to immunohistochemistry. *P < 0.05; **P < 0.01; ***P < 0.001; mean ± SD; *n* = 5. Ctrl, control; EGF, epidermal growth factor; EPSC-Exos, epidermal stem cell-derived exosomes; CD31, platelet endothelial cell adhesion molecule-1; IHC, immunohistochemical staining

Next, we clarified the effects of EPSC-Exos on nerve and vessel regeneration of damaged skin using IHC. The results showed that after 4 weeks of treatment (as shown in Fig. 4a–c), the skin of the EPSC-Exos group exhibited significantly more vessels (CD31^+^) and nerves (nestin^+^) than in the EGF and control groups (*P* < 0.001). Also, the proliferation (Ki67+) of skin cells in the EPSC-Exos group was also higher than in the EGF and control groups (Fig. 4d). These results suggest that EPSC-Exos can promote nerve and vessel regeneration and cell proliferation in wound healing.

### EPSC-Exos inhibit TGF-β1 expression from suppressing myofibroblast formation in vivo and in vitro

**Fig. 5:**
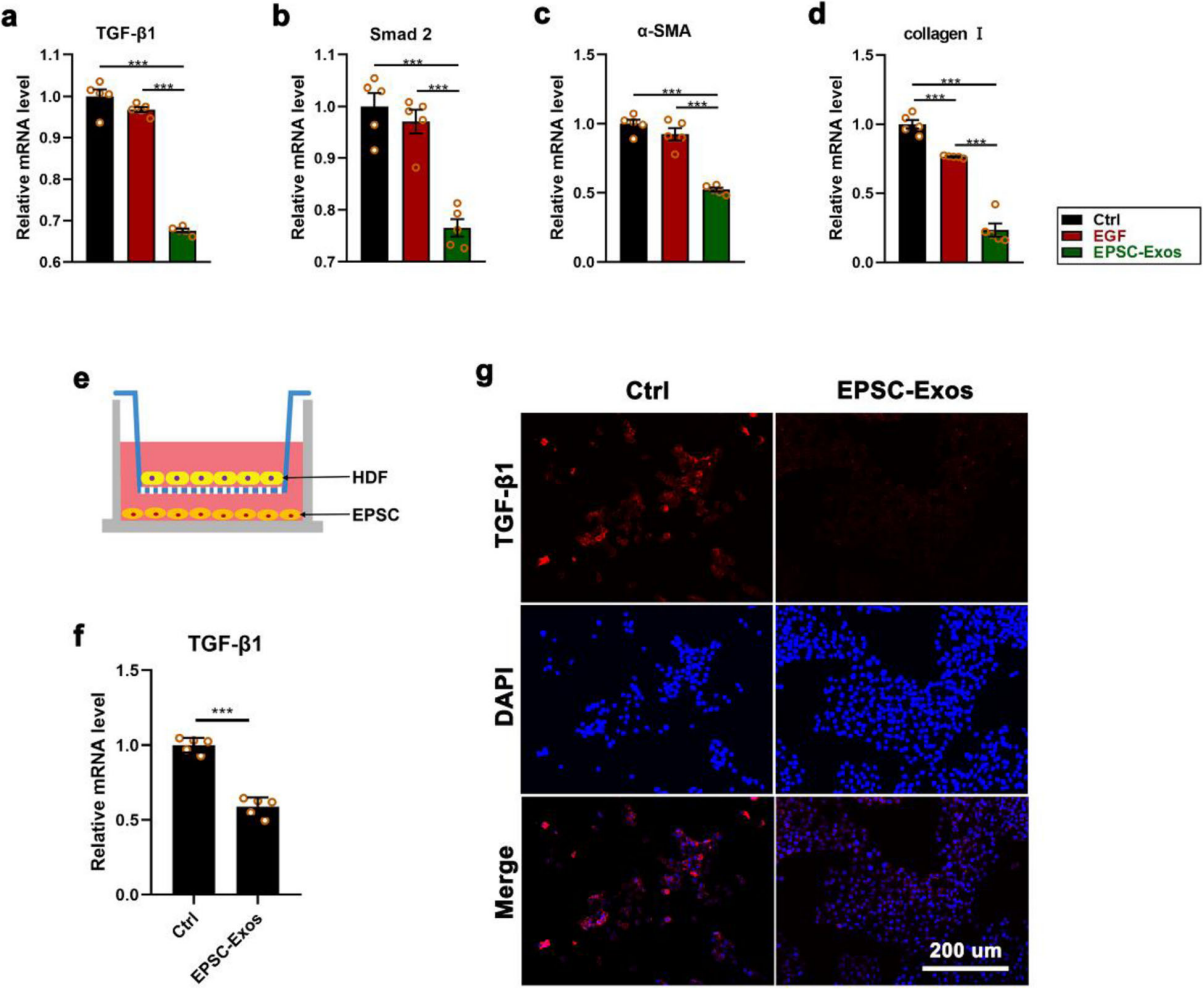
EPSC-Exos inhibited the expression of transforming growth factor-β1 (TGF-β1) and its downstream genes to suppress myofibroblast formation. **a**–**d** mRNA levels of TGF-β1, Smad2, α-SMA and collagen I in the healing tissue. **e** ESCs were co-cultured with HDF in Transwell plates (membrane well, 0.4 μm). IF and qRT-PCR were performed after culture for 48 h. **f**, **g** Expression levels of TGF-β1 of HDF in a co-cultured system using qRT-PCR and IF. Scale bar = 200 μm. ***P < 0.001; mean ± SD; *n* = 5. Ctrl, control; EGF, epidermal growth factor; EPSC-Exos, epidermal stem cell-derived exosomes; ESC, epidermal stem cell; HDF, human dermal fibroblast; TGF-β1, transforming growth factor β-1; α-SMA, α-smooth muscle actin; IF, immunofluorescence staining

TGF-β1 is the main factor in inducing dermal fibroblasts and the most important cell in skin wound healing and for differentiation into myofibroblasts [1, 3, 4]. Inhibiting TGF-β1 may be an essential means to reduce scar formation in wound healing. To explore the mechanism of EPSC-Exos inhibiting scar formation in wound healing, we measured the TGF-β1 expression levels in the healing tissue of rat models and HDF co-cultured with ESCs. The results showed that the mRNA levels of TGF-β1 and its downstream genes, including Smad2, α-SMA and collagen I, in the healing tissue of rats were all significantly lower than in the EGF and control groups (*P* < 0.001) (Fig. 5a–d). Also, expression levels of TGF-β1 were evaluated on HDF treated with EPSC-Exos, co-cultured with ESC in Transwell plates for 48 h (Fig. 5e). As shown in Fig. 5f and g, expression levels of TGF-β1 treated with EPSC-Exos were significantly reduced in mRNA and protein compared with the control group (*P* < 0.001). These results suggest that EPSC-Exos may inhibit the expression of TGF-β1 to suppress myofibroblast formation in wound healing.

### EPSC-Exos-specific microRNAs target TGF-β1 to suppress myofibroblast differentiation

**Fig. 6:**
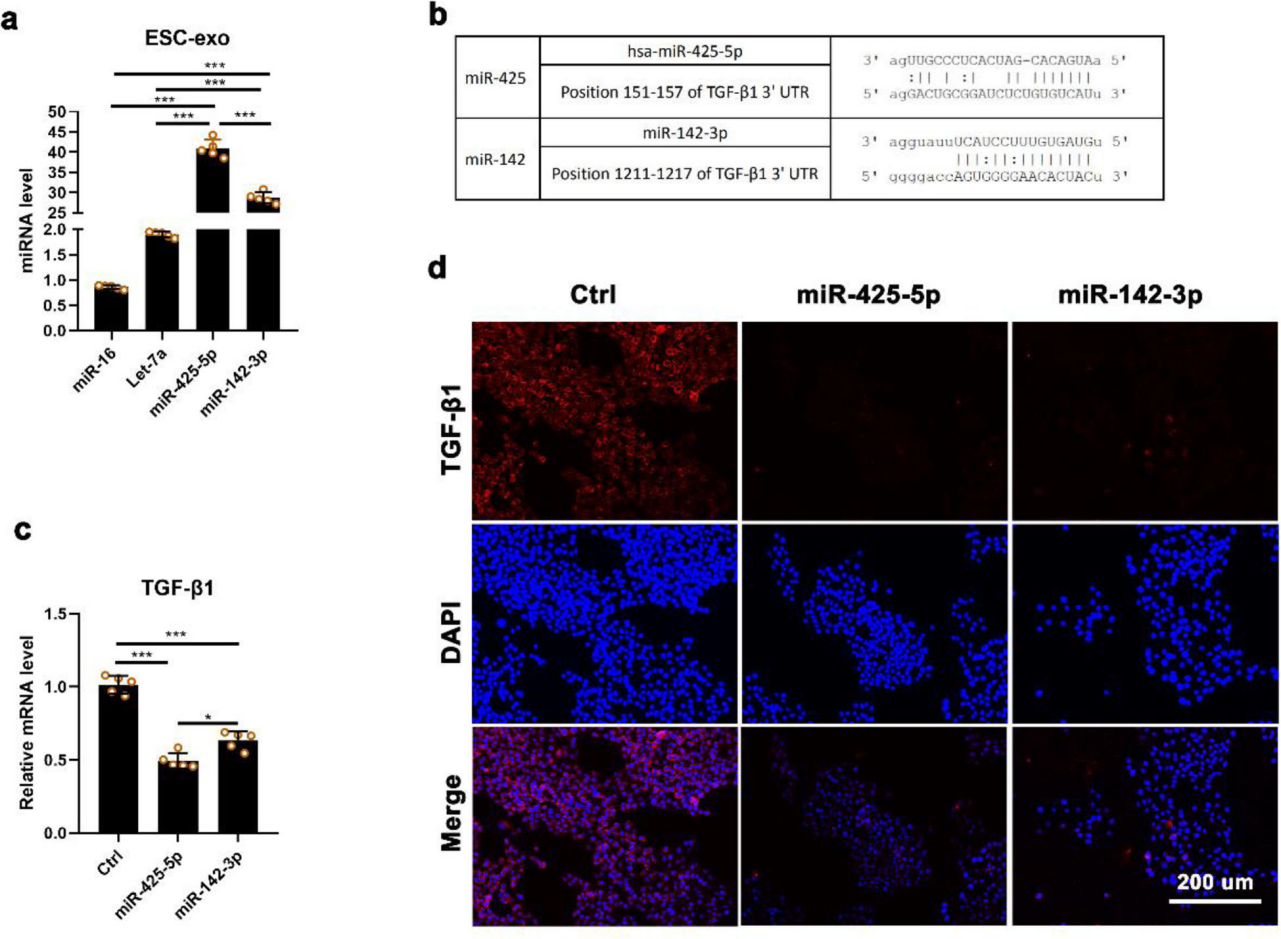
EPSC-Exos-specific microRNAs inhibited TGF-β1 expression of HDF. **a** Main miRNAs in EPSC-Exos using qRT-PCR. **b** A list of predicted binding sites of EPSC-Exos-specific miRNAs and their targets. **c**, **d** TGF-β1 mRNA levels of HDF treated with miR-425-5p agomir and miR-142-3p agomir of HDF, respectively. Scale bar = 200 μm. *P < 0.05; ***P < 0.001; mean ± SD; *n* = 5. Ctrl, control; EGF, epidermal growth factor; EPSC-Exos, epidermal stem cell-derived exosomes; HDF, human dermal fibroblast; TGF-β1, transforming growth factor β-1; IF, immunofluorescence staining

We found that miR-16, let-7a, miR-425-5p and miR-142-3p were highly expressed in EPSC-Exos detected using qRT-PCR (Fig. 6a). To predict the potential target genes of these microRNAs, we used TargetScan (http://www.targetscan.org/) and Microrna (http://www.microrna.org) for analysis. The results showed that miR-425-5p and miR-142-3p were all directly targeted to TGF-β1 (Fig. 6b) To verify the role of miR-425-5p and miR-142-3p in EPSC-Exos, we first added agomirs in HDF cultural system to test whether they can affect the expression of TGF-β1. The qRT-PCR and IF analysis showed that these 2 microRNAs significantly suppressed the expression of TGF-β1 (Fig. 6c, d). The results suggest that EPSC-Exos may inhibit the differentiation of fibroblasts to myofibroblasts via suppressing TGF-β1 expression via miR-425-5p and miR-142-3p.

## Discussion

Scar formation is an undesirable and significant result of both wound healing and fibrosing disorders. Excessive accumulation of myofibroblasts is a crucial factor for scar development, which can even lead to tissue or organ contraction [1, 4, 21]. Regulating myofibroblast formation may be an effective approach to preventing abnormal scarring. EPSC-based therapies have been proven to improve wound healing and reduce scar formation [22, 23]. Researchers have evaluated the effects of EPSC on tissue repair and wound healing. However, no previous studies have focused on the effects of EPSC-Exos on wound healing and scar formation. This study is the first to report that EPSC-Exos suppress myofibroblast differentiation in wound healing, which may be achieved by EPSC-Exos-specific microRNAs. Our research provides new insights for the application of EPSC-Exos to prevent scar formation.

EPSC, similar to adipose stem cells, are a cell type widely used in clinical research and regenerative medicine because they are numerous, are easy to obtain and have fewer ethical controversies than embryonic stem cells [22, 24, 25]. EPSC promote re-epithelisation, angiogenesis and hair growth in wound healing, as well as stimulate the recruitment and proliferation of endogenous stem cells [22, 23, 25]. We speculated that EPSC might play a major role in cell therapy through paracrine. Therefore, the paracrine functions of EPSC were the focus of this study. We observed that the healing skin in the EPSC-Exos group was smooth with less scarring, which was significantly lower than that in the control and EGF groups.

Furthermore, we confirmed that the expression levels of TGF-β1 were lower than in the control and EGF groups. TGF-β1 is reportedly a pivotal factor in simulating fibroblast differentiation into myofibroblasts [26, 27]. Therefore, intervention is required during wound healing to prevent excessive formation of myofibroblasts, rather than taking remedial measures after scar formation.

Exosomes have content that includes mRNAs, regulatory miRNAs, functional protein and signalling lipids. Thus, they involve cell signalling and cell-to-cell communication and they influence tissue responses to injury, infection and disease [14, 28]. In this study, we found that the primary function of promoting skin healing and reducing scar formation may be caused by EPSC-Exos-specific miRNAs. The EPSC-Exos-specific miRNAs inhibited myofibroblast formation, which might be related to the targeting of TGF-β1-targeting microRNAs. In our previous study, several highly expressed specific microRNAs derived from EPSC-Exos were identified, including miR-16, let-7a, miR-425-5p and miR-142-3p. miR-425-5p [29] and miR-142-3p [30] have been previously reported to suppress fibrotic diseases, and most studies have suggested that these two microRNAs directly target TGF-β1, consistent with our present findings. We believe the functions of microRNAs in different organs and tissues might be the same. Therefore, our study on the effects of EPSC-Exos on these two microRNAs might help us better understand microRNAs’ functions. We believe these EPSC-Exos miRNAs could be essential inhibitors of TGF-β1, reducing the myofibroblast formation during skin wound healing.

Exosome-derived microRNAs are more stable than their parental cells. They can resist degradation in the circulation process in the body through the vesicle structure released by the parental cells [18, 31]. In the subsequent production and application, we can use EPSC as a factory to produce exosomes, making EPSC produce more functional microRNAs via transfection. Compared with EPSC, EPSC-Exos might be safer and more efficient in clinical applications. They may also have other advantages, such as easier storage and production, a lower risk of side effects and easier quality control. Thus, we suggest that EPSC-Exos could be a candidate strategy for promoting healing and reducing scar formation in the future.

## Conclusions

**Fig. 7:**
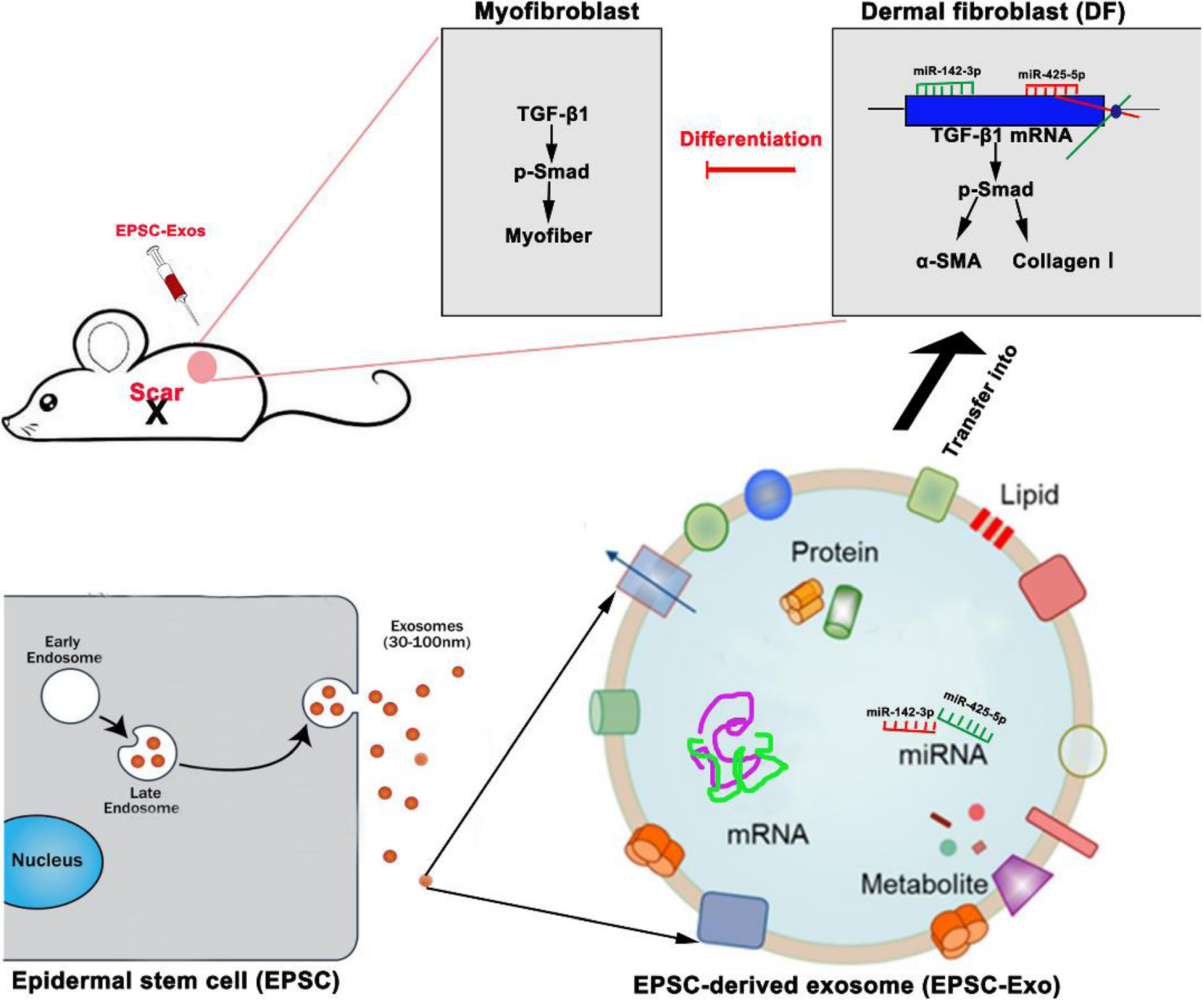
EPSC-Exos promoted wound healing and reduced scarring. MiR-425-5p and miR-142-3p carried by EPSC-Exos might suppress myofiber and collagen I deposition via downregulating TGF-β1 expression

This study has shed light on the specific microRNAs of EPSC-Exos and clarified a new approach for using stem cell therapy to promote wound healing and reduce scarring. miR-425-5p and miR-142-3p from EPSC-Exos might suppress myofiber and collagen I deposition via downregulating TGF-β1 expression (Fig. 7). As an alternative to cell therapy, EPSC-Exos might have a clinically beneficial anti-scarring effect.

## Supplementary information

**Additional file 1.**

## Data Availability

The datasets used and/or analysed during the present study are available from the corresponding author on reasonable request.

## Abbreviations

α-SMA: α-Smooth muscle actin
CD31: Platelet endothelial cell adhesion molecule-1
EGF: Epidermal growth factor
EPSC: Epidermal stem cells
EPSC-Exos: EPSC-derived exosomes
HDF: Human dermal fibroblasts
IF: Immunofluorescence staining
IHC: Immunohistochemical staining
PBS: Phosphate-buffered saline
TGF-β1: Transforming growth factor-β1
