# Supplementary material for: Epidermal stem cell-derived exosomes promote skin regeneration by downregulating transforming growth factor-β1 in wound healing
Source: Stem Cell Res Ther. 2020 Oct 23;11:452. doi: 10.1186/s13287-020-01971-6 (PMC7584097; doi:10.1186/s13287-020-01971-6)
Supplement: Supplementary file 1 — Additional file 1. [file 13287_2020_1971_MOESM1_ESM.docx]

Supplement

**Figure S1** Designed Primers

| **Gene name** |  | **Sequence** |
| --- | --- | --- |
| Collagen I | Forward | 5´- GTGAACGTGGTGAAGTTGGC -3’ |
|  | Reverse | 5´- TCACCACGACTTCCAACAGG-3’ |
| Collagen III | Forward | 5´- AGCCACCTTGGTCAGTCCTA -3’ |
|  | Reverse | 5´- GTGTAGAAGGCTGTGGGCAT-3’ |
| α-SMA | Forward | 5´-ATGGAGGGGAATACAGCCC -3’ |
|  | Reverse | 5´-ACTACTGCCGAGCGTGAGAT -3’ |
| Smad2 | Forward | 5’-AAGCCATCACCACTCAGAATTG-3’ |
|  | Reverse | 5’-CACTGATCTACCGTATTTGCTGT-3’ |
| TGF-β1 | Forward | 5’-GCCCTGGATACCAACTATTGCTTCA-3’ |
|  | Reverse | 5’-CAGAAGTTGGCATGGT-3’ |
| GAPDH | Forward | 5’-TGCCCCCATGTTTGTGATG-3’ |
|  | Reverse | 5’-TGTGGTCATGAGCCCTTCC-3’ |
| miR-16 | Forward | 5’-CGCAGTAGCAGCACGTA-3’ |
|  | Reverse | 5’-CAGTTTTTTTTTTTTTTTCGCCAA-3’ |
| Let-7a | Forward | 5'-GCCGCTGAGGTAGTAGGTTGTA-3' |
|  | Reverse | 5'-GTGCAGGGTCCGAGGT-3' |
| miR-425-5p | Forward | 5′-GGGGAGTTAGGATTAGGTC-3′ |
|  | Reverse | 5′-TGCGTGTCGTGGAGTC-3′ |
| miR-142-3p | Forward | 5′- GTGCAGGGTCCGAGGT-3′ |
|  | Reverse | 5′- ATCATAGAGGAAAATCCACG -3′ |
| U6 | Forward | 5′-CTCGCTTCGGCAGCACA-3′ |
|  | Reverse | 5′-AACGCTTCACGAATTTGCGT-3′ |
